# Large anterior congenital urethral diverticulum in an infant: a case report

**DOI:** 10.3389/fsurg.2024.1374168

**Published:** 2024-08-26

**Authors:** Mohammed A. Hassan, Rawa Bapir, Ismaeil Aghaways, Nali H. Hama, Mohammed Fahad Raheem, Wirya N. Sabr, Bilal A. Mohammed, Honar Othman Kareem, Ayman M. Mustafa, Fahmi H. Kakamad

**Affiliations:** ^1^ Urological Department, Sulaimani Teaching Hospital, Sulaimani, Iraq; ^2^ Scientific Affairs Department, Smart Health Tower, Madam Mitterrand Street, Sulaimani, Iraq; ^3^ Kscien Organization for Scientific Research (Middle East Office), Sulaimani, Iraq; ^4^ Department of Surgery, College of Medicine, University of Sulaimani, Sulaimani, Iraq

**Keywords:** congenital anterior urethral diverticulum, urethrocystoscopy, urinary tract infection, anterior urethral valve, management

## Abstract

**Introduction:**

A Urethral diverticulum can be defined as sac-like dilation lined with epithelial tissue, which may be congenital or acquired. It usually develops in the penoscrotal angle region but can also be observed in the penile urethra. It usually occurs in female teenagers. This report aims to discuss a male infant with a large urethral diverticulum.

**Case presentation:**

A 5-month-old male presented to the urological department at Sulaimani Teaching Hospital with a penile swelling that had been noticeable since birth. Clinical examination revealed a ventral cystic penile shaft swelling, which would fill with fluid during urination. A urethrocystoscopy was performed and showed a wide cystic ventral diverticulum. Diverticulectomy was performed as a surgical approach to remove the diverticulum.

**Discussion:**

Congenital anterior urethral diverticulum is an uncommon condition that typically begins in early life. It can manifest with various symptoms, like recurrent infections of the urinary tract, painful urination, and post-void urine dribbling. Diagnosis involves imaging, with urethrocystoscopy, to rule out other potential diagnoses. Different surgical techniques exist that show promising results in preventing recurrence. The current case involved diverticulectomy and multi-layered wound closure with a dartos flap.

**Conclusion:**

Large anterior diverticulum in early infancy is rare but possible; operation is the preferred intervention method.

## Introduction

A urethral diverticulum can be described as a sac-like dilation lined with epithelial tissue that is distinct from the urethra but connects to its inner space via a specific opening (1). These are rare occurrences in male children, manifesting either congenitally or acquired, with acquired cases being more prevalent (2, 3). It is typically developing at the early onset of life but can occur at any time, with the average age of onset being around 13 years old (4, 5). It was first identified by Watts in 1906 (2).

The clinical presentation varies depending on the patient's age and the urinary obstruction. Typical symptoms in adults may include diminished urinary flow, leakage after urination, recurrent urinary tract infections (UTIs), and penile swelling ventrally. While in neonates and infants, CAUD frequently displays non-specific symptoms like fever, diarrhea, and vomiting (6). A congenital anterior urethral diverticulum (CAUD) diagnosis is made through obstructive lower urinary tract symptoms, often accompanied by a penoscrotal mass. Typically, this diagnosis is confirmed through retrograde urethrogram or Micturating Cystourethrogram (2). It can be challenging to differentiate CAUD from anterior urethral valve (AUV) due to their causal relationship. The diverticulum linked to AUV is generally regarded as a non-authentic diverticulum since, in CAUD, there is an acute angle primarily between the proximal part of the expanded area and the ventral floor (penoscrotal angle region). In contrast, this acute angle is absent in AUV (7, 8).

Anterior congenital urethral diverticulum often develops in areas with a deficiency in the Corpus cavernosum urethrae, resulting in a thin-walled urethra (9). Treatment decisions depend on symptom severity, diverticulum size, and associated upper urinary tract changes. Observation may suffice for mild cases, while more severe cases may necessitate endoscopic or open surgical removal (1). Controversy exists regarding the surgical approach for managing the condition, which involves performing diverticulectomy with or without urethroplasty based on the size of the developed diverticulum and the endoscopic approach (2). In this study, a case of CAUD in a 5-month-old boy is reported, along with a discussion on the disease's presentation and management.

## Case presentation

### Patient information

**Figure 1:**
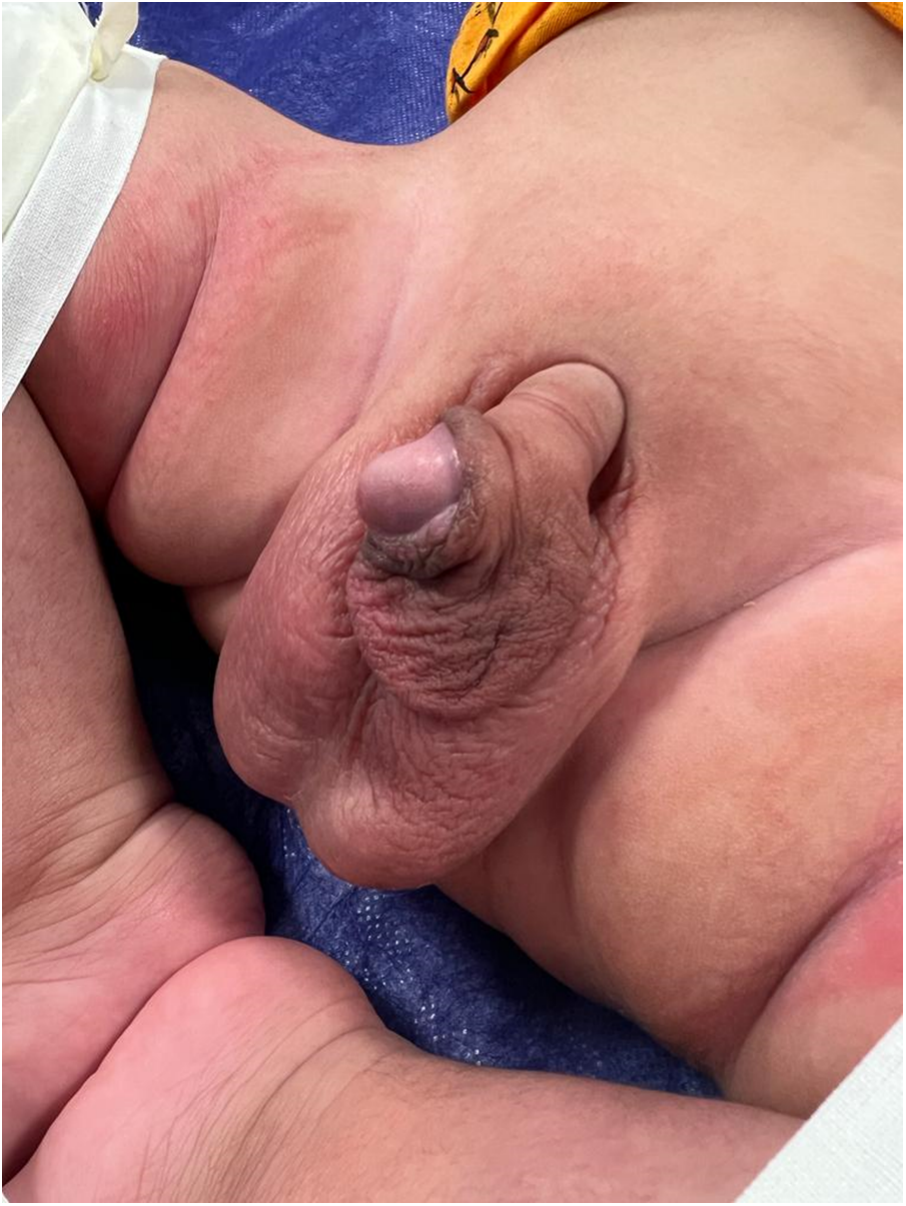
Shows a skin flap over the ventral surface of the penis.

A 5-month-old male presented to the Sulaimani Teaching Hospital with a penile swelling that had been noticeable since birth (Figure 1). The swelling had gradually increased and appeared to grow as the baby continued to develop, especially during micturition.

### Clinical findings

Clinical examination revealed a ventral cystic penile shaft swelling, which would fill with fluid during urination.

### Diagnostic approach

To further evaluate the patient's condition, a urethrocystoscopy was performed. This diagnostic procedure revealed a large ventral urethral diverticulum located approximately 1.5 cm proximal to the fossa navicularis. The defect due to the diverticulum was estimated to be about 2 cm, which helped confirm the diagnosis.

### Therapeutic intervention

**Figure 2:**
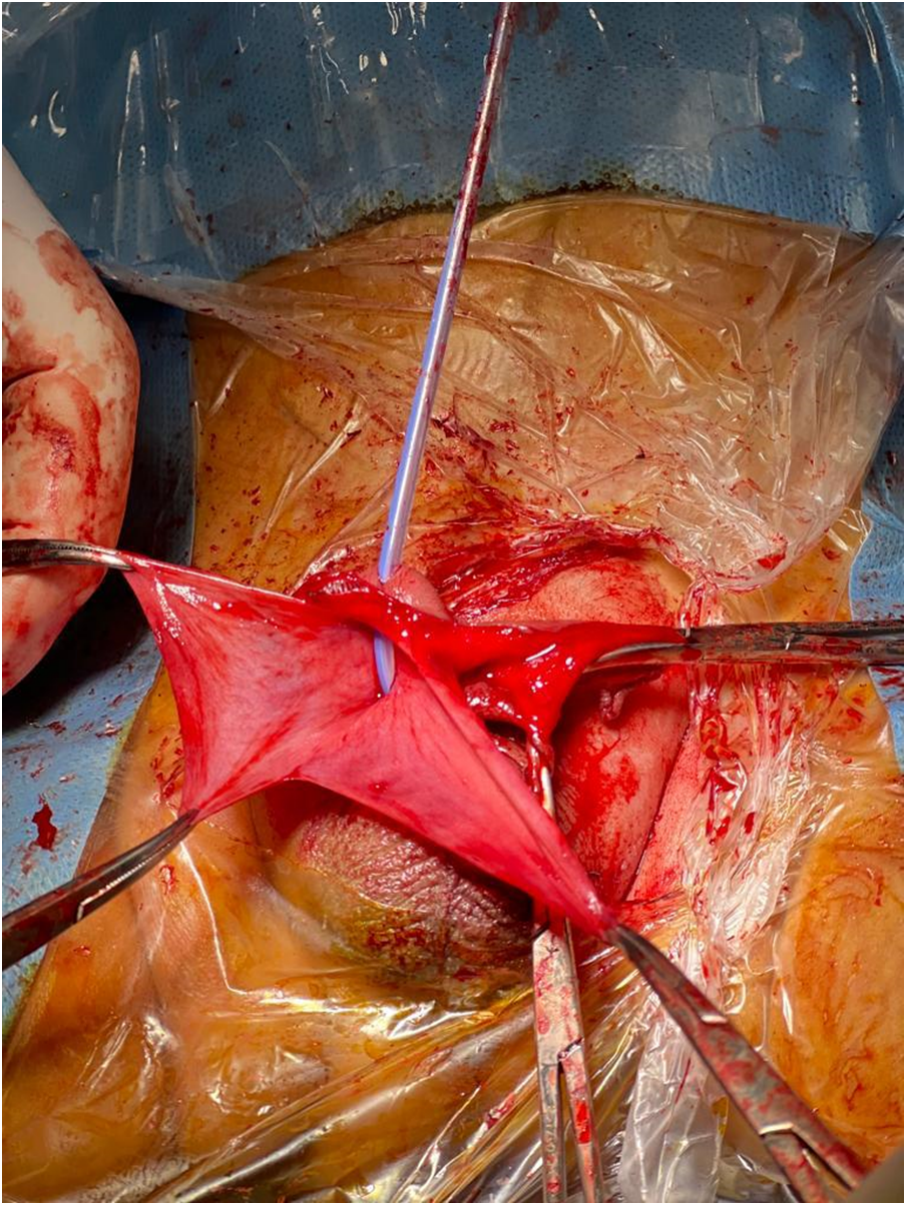
Intra-operative picture showing walls of the diverticulum.

**Figure 3:**
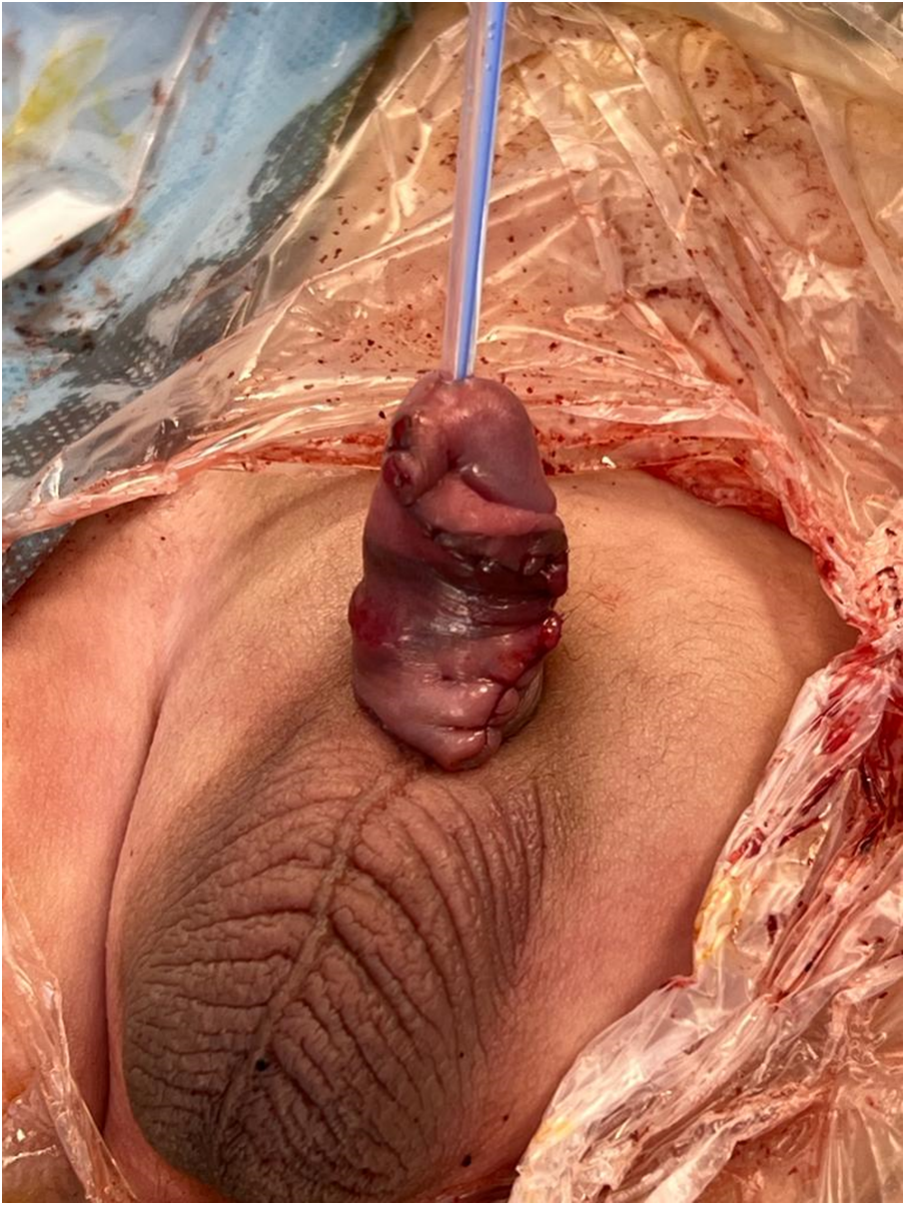
The post-operative appearance of the penis after diverticulectomy and closure.

Under general anesthesia, the patient was placed in a supine position; a Foley catheter was inserted. A vertical ventral penile shaft incision was made at the site of the bulging area. During the procedure, the surgical team dissected through the layers of skin, dartos fascia, areolar tissue, and penis fascia. This dissection identified a very thin corpus spongiosum, dissected further until the urethral diverticulum was reached and opened vertically. Stay sutures were placed at the edges of the diverticulum wall (Figure 2). The diverticulum was removed and the urethra sutured, then a dartos flap was utilized to cover the suture line of the urethra (Figure 3).

### Postoperative outcome

A 4-month follow-up revealed an uneventful recovery, with the suture line well-covered by a dartos flap.

## Discussion

The cause of CAUD is not yet completely understood, although several theories have been suggested to explain its formation (3). One theory suggests that they develop due to a partial absence or absence of the spongy body, often associated with hypospadias that led to urethral dilation. Another theory proposes that during embryogenesis, the urethral diverticulum forms due to epidermal pockets that communicate with the ventral urethral wall. As the anterior urethral tube develops, congenital cysts may form within the urethral groove, potentially leading to the formation of a diverticulum through the spontaneous rupture of these cysts into the urethral lumen (1, 3).

Congenital urethral diverticulum in males is an uncommon condition. The number of reported cases among children is currently unknown. Over the past two decades, 260 cases have been reported in the genuine literature, with no distinct separation between AUV and CAUD (1, 7, 10). In the current study, the examination revealed cystic swelling in the penile shaft ventrally that had increased in size during urination; this condition had been noticeable since birth.

A thorough examination of a child's medical history will uncover that these children have experienced consistently poor urinary flow since birth, and a clear indicator of this issue is the presence of cystic swelling in the urethra of the penis (4). This is consistent with the condition explained in the current study.

Diagnosis of AUD or CAUD is typically established through MCUG (Micturating Cystourethrogram) or retrograde urethrogram, which is sufficient for directing the diverticulum's location, determining its size, and identifying any associated problems, such as urethral stenosis. The MCUG involves filling the bladder with contrast dye during urination to observe the flow and identify diverticulum morphology, providing dynamic imaging that highlights functional aspects of the urethra.

Retrograde urethrogram, on the other hand, involves injecting contrast dye directly into the urethra to visualize its anatomy and identify specific diverticula. This technique is particularly useful for detailed anatomical mapping and precise measurement of diverticulum size, aiding in surgical planning. Furthermore, in cases where uncertainty exists regarding alterations in anatomy resulting from the urethral diverticulum, additional imaging methods like magnetic resonance imaging (MRI) may be required. It is particularly valuable for assessing soft tissue structures, such as identifying the extent of diverticulum involvement, evaluating adjacent organs, and detecting any associated anomalies or complications that may impact surgical management (2, 8). Furthermore, diverticula associated with anterior urethral valves (AUV) differ from true diverticula. These diverticula cause proximal deformation and dilation of the urethra. Additionally, syringocele of Cowper's duct can contribute, as it often communicates directly with the gland. Hence, these formations are termed pseudodiverticula (3, 11).

During surgery, intraoperative urethrocystoscopy enables direct observation of the diverticulum, which appears as a urethral dilatation of varying dimensions, characterized by the presence of the urethral mucosa and a connection through an opening to the urethral lumen. Babty et al. recommended urethrocystoscopy as a diagnostic approach for CAUD (2). In the current study, urethrocystoscopy was also used so that the patient could thoroughly examine both the urethra and bladder, and rule out other potential diagnoses.

There is controversy regarding the management of CAUD. A small AUD can be addressed through distal lip removal by transurethral endoscopy (6). A large diverticulum, like the one in this case, or a diverticulum with stones should be managed by removing the diverticulum and restoring the urethra (12). Continuity can be established through either an anastomosis or a urethroplasty procedure. In a study by Alphs et al., 13 symptomatic urethral diverticula were treated surgically from 2003 to 2008. Diverticulum removal with a primary anastomosis was done for urethral defects measuring less than 4 cm. In comparison, for urethral defects measuring 4 cm or more, substitution urethroplasty was done, both with the same outcome (13). Furthermore, another study proposed a different approach for three children undergoing hypospadias repair, which involved excising the diverticulum and closing the urethra by incorporating overlapping suture lines (14). Additionally, Ronzoni et al. proposed another approach: the Monseur technique. In this adjusted method, the procedure included separating the urethra from the corpora cavernosa, spanning at least 10 cm, and creating a full-thickness incision in an “italic S” shape on the ventral side of the urethra. The urethra was subsequently rotated by 180 degrees along its length, and its edge was sutured to the tunica albuginea of the corpora. Out of 48 patients, none of them had a reoccurrence of the diverticulum (15). Allen et al. also conducted a study on 21 patients, with 7 having congenital defects and 14 having acquired ones. A surgical intervention was performed on 19 out of 21 patients (90%). The surgical approach included primary excision and repair. During an average follow-up period of 60 months, they documented postoperative complications in 64% of patients with acquired-type diverticulum and 27% of patients with congenital-type diverticulum (16). According to Quoraishi et al., an endoscopic approach was used to incise the lip of the diverticulum. However, since the diverticulum pouch still exists, it may develop a flap again, which may require repeat procedures. This can result in scar tissue formation and a urethral stricture (17). Generally, the outcome was good among all cases using diverticulectomy and urethroplasty, as in the current case, with a follow-up period between three months and two years (3, 12). Comparatively, primary anastomosis is preferred for smaller defects due to its lower invasiveness and faster recovery, while substitution urethroplasty is reserved for larger defects where more extensive reconstruction is needed. The Monseur technique and endoscopic approaches, though effective in specific cases, highlight the diversity of surgical options tailored to the complexity of the diverticulum, reinforcing the need for individualized treatment plans.

In the current case, a diverticulectomy was performed, which involved removing the diverticulum. The surgical wound was then closed carefully in layers, and a dartos flap was utilized in the closure process.

## Conclusion

Large anterior diverticulum in early infancy is rare but possible; operation is the preferred intervention method.

## Data Availability

The original contributions presented in the study are included in the article/Supplementary Material, further inquiries can be directed to the corresponding author.
